# Preparation of transgenic Iranian lizard Leishmania coding HIL-12

**Published:** 2017-10

**Authors:** Tahereh Donyavi, Mojgan Bandehpour, Bahram Kazemi

**Affiliations:** 1 Departement of Biotechnology, School of Advanced Technologies in Medicine, Shahid Beheshti University of Medical Sciences, Tehran, Iran; 2 Cellular and Molecular Biology Research Center, Shahid Beheshti University of Medical Sciences, Tehran, Irann; 3 Departement of Biotechnology, School of Medicine, Shahid Beheshti University of Medical Sciences, Tehran, Iran; 4 Vice Chancellor for Health, Iran University of Medical Sciences, Tehran, Iran

**Keywords:** hIL-12, Iranian lizard Leishmania, Cysteine peptidase C, Leishmanization

## Abstract

**Background and Objectives:**

Leishmania are intracellular flagellate protozoan parasites cause a wide spectrum of clinical manifestations in human. The immunological basis for resistance against leishmaniasis depends on Thl responses in the course of performance of cytokines like IL-12. In this study, a transgenic Leishmania coding human IL-12 was produced that can be used in Leishmanization.

**Materials and Methods:**

A fragment of Iranian lizard Leishmania (I.L.L) gene, named Cysteine Peptidase C (CPC), was amplified separately as two parts with PCR reaction. Then, they were attached using SOEing PCR such that the restriction site of *SalI* was placed in the middle of it. SOEing PCR product was purified and cloned in *HindIII* restriction site of pGEM-7z-f and named pKDB-CPC. After clone optimization, the hIL-12 construct was cloned in *SalI* restriction site of pKDB-CPC and named pKDB-IL12. Prokaryotic section of the above construct was removed and transferred into I.L.L by electroporation.

**Results:**

Production of recombinant hIL-12 in transgene parasites was proved by ELISA. rhIL-12 secreted into supernatant culture medium accumulated at concentrations up to 246.53 ± 15.92 pg.mL^−1^.

**Conclusion:**

Targeted gene replacement into the I.L.L genome using plasmid pKDB-cpc identical replacement process was successfully completed for the first time. Stabilized recombinant DNA consist of target gene didn’t have any toxicity for the parasite. Transgenic I.L.L produced and secreted active human interleukin 12 and can be an appropriate candidate for Leishmanization.

## INTRODUCTION

Leishmaniasis is a common neglected infectious disease in many tropical and subtropical parts of the world (1), which is transmitted to humans by the bite of infected female sandfly. There are three forms of disease: cutaneous, mucocutaneouse and visceral. Visceral leishmaniasis, also known as Kala-azar is fatal if left untreated. Increased incidence of leishmaniasis has been caused by climate change, population mobility, AIDS epidemic (2), wide use of immunosuppressive drugs and war. According to the world health organization (WHO) in June 2016 from 11 countries with high prevalence of this disease, 399 million cases of cutaneous leishmaniasis and 556 million cases of visceral leishmaniasis has been occurred. These 11 countries are: Afghanistan, Algeria, Brazil, Colombia, Iran, Syrian Arab Republic, Bangladesh, Ethiopia, India, South Sudan and Sudan. The current strategic framework to control leishmaniasis is based on chemotherapy and personal protection. Research and development for a safe, effective and affordable anti leishmania vaccine is one of the WHO priorities. Anti leishmania vaccine trials are focused on: dead parasite of leishmania major, subunit vaccines with adjuvants (3) or surface protein of leishmania parasite and its recombinant peptides (4). Although the live-attenuated strains of pathogenic parasites in leishmaniazation mimic the immunogenicity path and stimulation of immune response in a way which makes effective vaccine production promising, it is always that in immune deficient people and those infected by HIV these strains return back to virulence. Therefore, there is no effective vaccine against any types of leishmaniasis and much interest has been arisen in development of vaccine by utilizing non-pathogenic (5) and DNA vaccination (3) in leishmanization. *Leishmania tarentolae*, as a non-pathogenic strain in mammals, which was found in Brazilian mummy (6) grows rapidly in a culture media at low cost and is very similar to mammalian cells in protein expression, post-translational modifications and glycosylation pattern (7). It also resembles *Leishmania* major regarding antigenicity and life-cycle in mammal macrophage. It can stimulate the host cellular immune system such that its injection to Balb-c mice creates immunogenicity effects against the pathogenic strain (5). Shahbazi et al. in a recent study vaccinated outbreed dogs with a recombinant *L. tarantula* expressing the *L. donovani* A2, CPA and CPB antigens. They demonstrated the preventive effects of recombinant *L. tarentolae* A2-CPA-CPB as a live non-pathogenic vaccine against challenge pathogenic strains (8). On the other hand, the decisive role of IL-12 in the successful start of the host’s defense against visceral leishmaniasis has been proven in Balb/c mice (9). Also, IL-12 has recently been used as the adjuvant in vaccination against *Mycobacterium tuberculosis* to increase cellular immune performance, and the effects of increased cellular immunity response have been recorded (10). This research was carried out based on previous knowledge in the field of recombinant *L. tarentolae*, IL-12 expression in eukaryote host and leishmanization. The aim of this study was to produce transgene non-pathogenic leishmania with IL-12 using homologous recombination in locus Cysteine Peptidase C (CPC) of parasite. It can be used in leishmanization. It must be noted that a new vector was designed and used to transfer the IL-12 gene into the Iranian lizard leishmania parasite genome.

## MATERIALS AND METHODS

### Materials and Methods.

Iranian Lizard Leishmania (I.L.L) isolated by Kazemi et al. (11) was present in the biotechnology lab. pGEM-7zf vector (Promega Company, USA) and pTG19-T vector (Vivantis, Malaysia) were purchased.

### Designing the construct containing human IL-12 gene.

The alpha chain gene sequence of human Interleukin 12 (GenBank accession number NM_000882.3), beta chain gene sequence of human Interleukin 12 (GenBank accession number NM_002187), CMV promoter gene sequence (GenBank accession number KJ361976.1), neomycin phosphotransferase gene sequence (GenBank accession number DQ789393.1) were received from NCBI data bank. Signal peptide gene sequence and Poly A tail of leishmania major (12) were added to human Alpha and Beta chain sequence of hIL-12. All Genes sequence were analyzed using webcutter 2.0 in regard to restriction enzyme cutting sites. *KpnI, XhoI, BamHI, EcoRI, SalI* restriction sites were placed on different parts of the construct. The final construct with 4207 base pairs was synthesized in pGH vector (named pGH-hL12) by Gene Ray Biotechnology Company (China).

### Cultivation and preparation of Iranian lizard leishmania (I.L.L).

Promastigotes of Iranian Lizard Leishmania (I.L.L) were cultivated in complete RPMI 1640 (GIBCO, US) containing 10% fetal bovine serum, 100 units/mL penicillin and 100 μg/mL streptomycin (GIBCO, Pen-Strep15140) at 23°C under shaking condition (110 rpm) and sub-cultured by diluting suspension 2–3 fold in fresh media every two days. When the promastigotes reached 3×10 ^8^ mL ^−1^ , there was enough to extract Leishmania genomic DNA.

### Extraction of I.L.L genomic DNA.

Genomic DNA of I.L.L was prepared using the TELT method (13).

### Designing and cloning of homologous arms.

For designing the homologous arms, 650 bp of Cysteine Peptidase C gene (CPC) of Parrott-TarII *Leishmania tarantula* was taken from GeneBank under accession number Ltap29.0870 with 1020 bp (14). It was analyzed with webcutter 2.0. The fragment of CPC gene sequence was used as a template to synthesis the homologous arms by adding *HindIII* restriction site at up and downstream. The two parts of CPC gene (229 and 417 base pairs) were amplified separately using LTa1, LTa2 and LTa3, LTa4 primers. PCR products were gel-purified with extraction from agarose using Qiaex II Gel purification kit (Qiagen) and attached to each other by SOEing PCR so that *SalI* restriction site was placed in the middle of the product. It was named Iranian Lizard Leishmania Cysteine Peptidase C (I.L.L-CPC). I.L.L-CPC was cloned into the pTG19-T and transferred into *E. coli* TOP 10 strain (15). Cloning confirmation was performed with PCR using M13R, M13F primers and sequencing.

The recombinant vector (pTG-19T-CPC) was digested by *HindIII* (Ferments, Lithuania) and purified by QIAquick Gel Extraction Kit (Qiagen) then sub-cloned into the *HindIII* site of pGEM-7zf CPC. It was named pKDB-CPC. pKDB-CPC was transformed into *E. coli* TOP 10 strain. Cloning confirmation of CPC gene was confirmed by PCR and restriction analysis using *HindIII* (Ferments, Lithuania).

### Cloning of human IL-12 construct into pKDB-CPC.

pKDB-CPC and pGH-hL12 were digested with *SalI* (Fermentas, Lithuania). The hIL-12 gene was extracted from the pGH-hL12 and purified by QIAquick Gel Extraction Kit (Qiagen) then cloned into *SalI* site of pKDB-CPC. It was named pKDB-IL12. pKDB-IL12 was transformed into *E. coli* TOP 10 strain. Cloning confirmation of hIL-12 gene was confirmed by PCR and restriction analysis using *HindIII*.

### Transfection of Iranian Lizard Leishmania (I.L.L).

10 ^8^ Promastigotes of I.L.L (11) were washed with electroporation buffer (Eppendorf, Germany) and suspended in 1 ml of electroporation buffer. The recombinant pKDB-IL12 was purified by Plasmid Miniprep Kit (Bioneer, Korea) and digested with *HindIII* and the pure expression cassette was isolated by QIAquick Gel Extraction Kit (Qiagen).

**Fig. 5.**
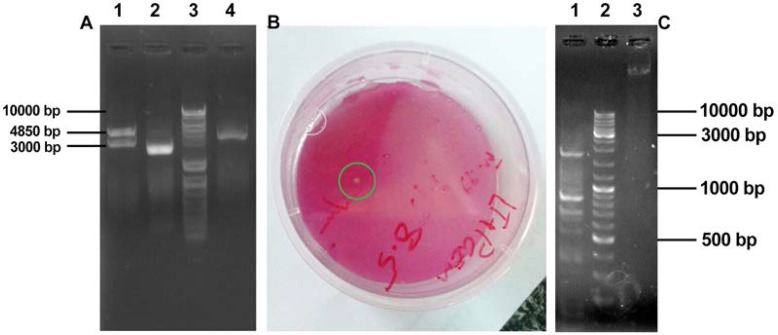
Panel A: pKDB-IL12 restriction analysis; lane1, *HindIII* digested pKDB-IL12; lane 2, *SalI* digested pKDB-IL12 (4200bp); lane 3, DNA size marker 100–10000bp (Fermentas, Lithuania); lane 4, uncut pKDB-IL12 Panel B: A colony of transgenic Iranian Lizard Leishmania with hIL-on solid media M199 Panel C: Confirmation of transgenic I.L.L with hIL-12. lane1, PCR product of transgenic ILL with hIL-12 using LTaF1 and LTaR1 primers (900 bp); lane 2, DNA size marker 100–10000bp (Fermentas Lithuania); lane 3; PCR reaction of Wild type ILL

50 μl of linear DNA including 5 μg DNA was added to 450 ml of I.L.L cell suspension containing 10 ^8^ cells ml ^−1^ in a 4mm cuvette and incubated on ice for 10 minutes. Electroporation was performed in a Multiporator (Eppendorf, Germany). The condition for the experiment was two pulses 2000V and 10 second interval time (16). Transfectants I.L.L were inoculated on RPMI 1640 media containing 10% fetal bovine serum, 100 units mL ^−1^ penicillin and 100 μg/mL streptomycin (GIBCO, Pen-Strep15140) at 23°C under shaking condition (110 rpm) for 24 hours. Stable transfectants were selected on RPMI 1640 media containing 25μg/ml neomycin (G418) after 48 hours and stringent selection was continued by increasing the concentration neomycin up to 100μg/ml for a week. For investigating evaluation of the expression cassette into the CPC locus of I.L.L, 1.5 mL aliquot of culture was subjected to genomic DNA extraction and diagnostic PCR was performed using hIL-12 reverse primer, LTaR1, and LTaF1 forward primer (Table 1) which located in the I.L.L CPC gene (annealing temperature 53°C) (Fig. 5).

**Table 1.** Primer sequences used in this study

**Primer name**	**Sequence (5′ to 3′)**
LTa1	5′ TTAAGCTTAATGGTCAGTGGTCCGCCTCGGC 3′
LTa2	5′ GGCGATCCGTCGACATGTTAGACATGG 3′
LTa3	5′ CCATGTCTAACATGTCGACGGATCGCC 3′
LTa4	5′ TTAAGCTTCTCGAAGGGGCCGTTGGTCATGAG 3′
LTaF1	5′ GTCTTTTACCAGGTCTGTGCTGTGCC 3′
LTaR1	5′ TGCGCGCTGGACACATGCTG 3′
M13 F	5′ GTAAAACGACGGCCAGTG 3′
M13R	5′ GGAAACAGCTATGACCATG 3′

As well as transfectants were selected on solid culture media containing neomycin (G418) and incubated for 10 days at 23°C for an emergence of colonies.

### ELISA and determination of human recombinant IL-12 concentration.

Expression of rhIL-12 in transfectants I.L.L was evaluated by IL-12 p70 DuoSet ELISA kit DY1270-05 (R&D system). Transfectant I.L.L were cultivated on RPMI 1640 media containing 100 units/ml penicillin and 100 μg/ml streptomycin (GIBCO, Pen-Strep15140). 1ml of culture media at 3, 7, 24 and 48 hours after cultivation was centrifuged at 1500 rcf for 10 min at 4°C. The supernatant was immediately aliquoted and stored at −80°C. Serial dilution was prepared from standard hIL-12 and the supernatant of the culture medium of transfectants and wild-type promastigotes and added to wells of ELISA plate coated with the human anti-IL-12 primary antibody. ELISA plate was washed and subjected to conjugated secondary antibody and detected by TMB. Absorption was measured at 450 nm wavelength and 630 nm reference wavelengths.

## RESULTS

### Preparing homologous arms (I.L.L CPC).

**Fig. 1.**
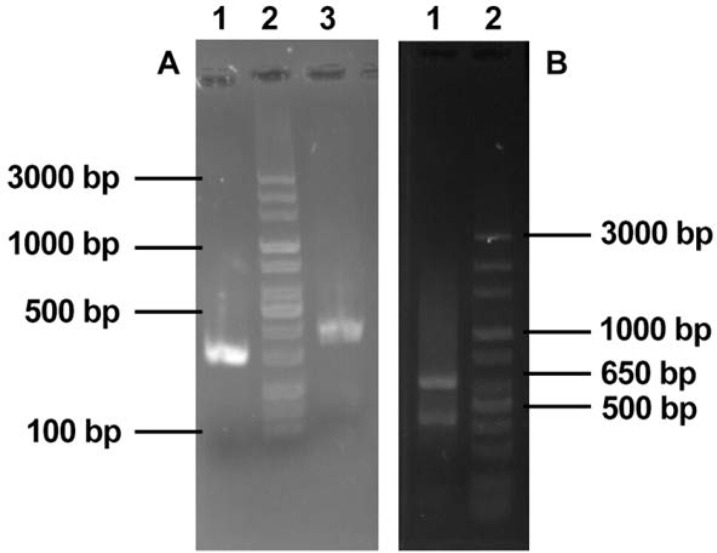
Panel A: Two fragments of ILL Cysteine Peptidase C (CPC); lane1, ILL CPC gene PCR product using LTa1 and LTa2 primers (229bp); lane 2, DNA size marker 100–3000bp (Fermentas, Lithuania); lane3, ILL CPC gene PCR product using LTa3 and LTa4 primers (417bp) Panel B: SOEing PCR product of two fragments of ILL Cysteine Peptidase C (ILLCPC); lane1, SOEing PCR product using LTa1, LTa4 primers (650bp); lane 2, DNA size marker 100–3000bp (Fermentas, Lithuania)

Two fragments of I.L.L Cysteine Peptidase C gene (I.L.L-CPC) containing 229bp and 417bp were amplified by LTa1, LTa2 and LTa3, LTa4 primers from I.L.L genomic DNA. PCR products were gel-purified and attached to each other by SOEing PCR (I.L.L-cpc (650bp)) (Fig. 1).

### Cloning of homologous arms into pTG-19T.

**Fig. 2.**
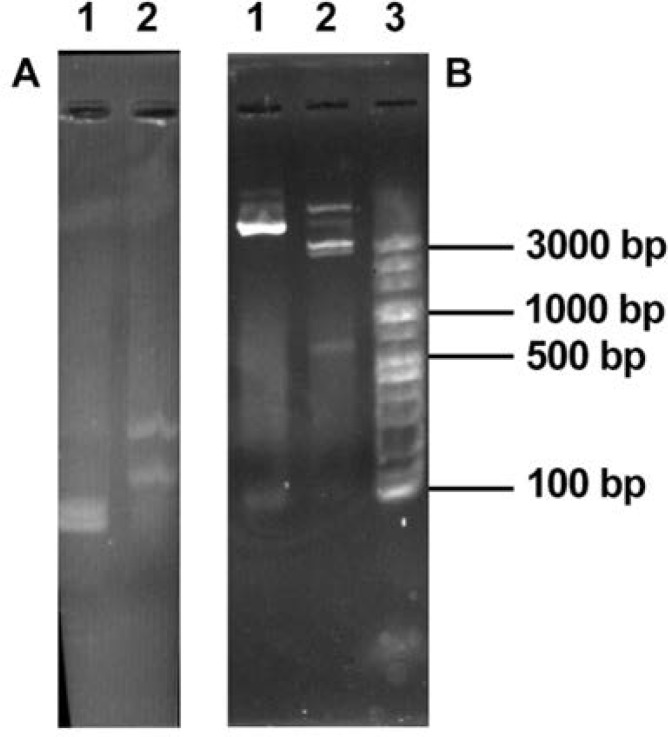
Panel A: *SalI* Digested I.L.L-CPC SOEing PCR product; lane1, ILL CPC SOEing PCR product (650bp), lane 2; ILL CPC SOEing PCR product digested by *SalI* wich resulted from 229bp and 417bp fragment Panel B: *HindIII* Digested recombinant pTG-19T I.L.L cysteine peptidase C (I.L.L-CPC); lane 1, Uncut pTG-19T I.L.L-CPC; lane 2, digested pTG-19T I.L.L-CPC (desired fragment 650bp); lane 3, DNA size marker 100–3000bp (Fermentas, Lithuania)

SOEing PCR product was ligated into pTG-19T. Cloning was confirmed by restriction enzyme digestion and PCR using M13F, M13R primers. The PCR product was sequenced and deposited into Gene Bank at accession number LC101919 (Fig. 2).

### Cloning of homologous arms into pGEM-7z-f.

**Fig. 3.**
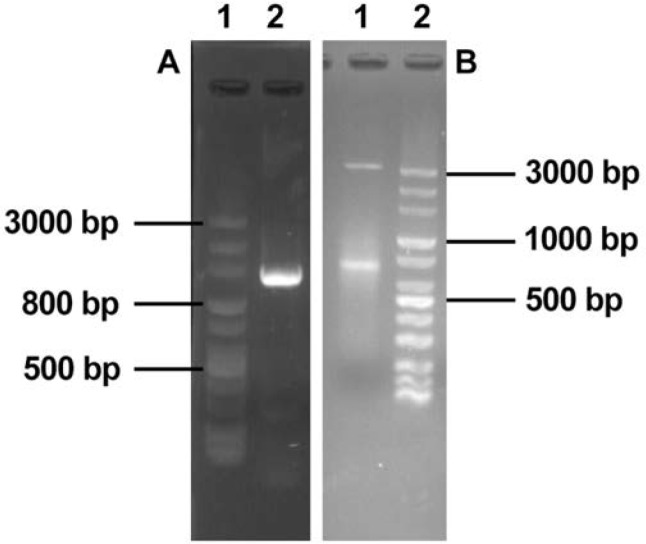
Panel A: Colony PCR of recombinant pTG-19TI.L.L- CPC; lane1, DNA size marker 100–3000bp (Fermentas Lithuania); lane 2, PCR product of recombinant pTG-19T-I.L.L-CPC using primers M13 forward & M13 reverse (850 bp) Panel B: *HindIII* Digested recombinant pKDB-cpc lane 1, After 3 hours I.L.L-CPC (650bp); lane 2, DNA size marker 100–3000bp (Fermentas Lithuania)

I.L.L-CPC gene fragment was ligated into *HindIII* restriction site of pGEM-7z-f (pKDB-CPC) and confirmed by restriction enzyme digestion (Fig. 3).

### Ligation of human IL-12 construct into pKDB-CPC.

**Fig. 4.**
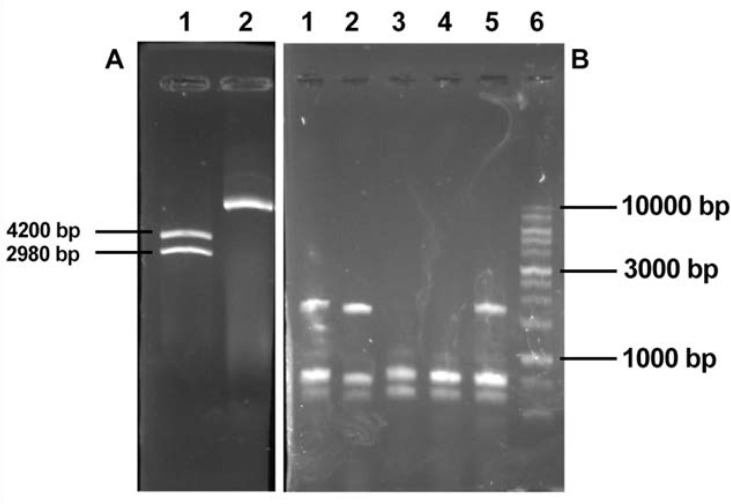
Panel A: *SalI* Digested pGH-hL12 (4200 bp); lane 2, uncut pGH-hL12 Panel B: Colony PCR of pGEM-7zf-ILLCPC; Lane1, 2, 5 positive bacteril colony PCR using LTa1, LTa4 primers; lane 3, negative control pGEM-7zf PCR product using primers LTa1, LTa4; lane 4, negative colony PCR product; lane 6 DNA size marker 100–10000bp (Fermentas, Lithuania)

pGH-hIL12 was digested with *SalI* and released hIL-12 gene constract. It was purified and subcloned into *SalI* restriction site of pKDB-CPC (pKDB-IL12). Gene cloning was confirmed through specific PCR reaction on hIL-12 gene and analyzed by *HindIII* and *SalI* restriction enzymes (Fig. 4).

### Transfection and selection of recombinant I.L.L. promastigotes.

After electroporation, Iranian Lizard Leishmania was successfully transfected as selected on solid culture medium M199 containing antibiotic. Transgenic colonies were appeared on plate agar after 10 days (Fig. 5).

### Human recombinant IL-12 produced by transgenic I.L.L.

hIL-12 in some 40 samples including supernatant of transgenic I.L.L promastigotes culture medium as positive samples, and wild type I.L.L promastigotes as negative samples were measured using ELISA. Production and secretion of rhIL-12 in the supernatant culture medium of I.L.L after 24 and 48 hours cultivation was confirmed. rhIL-12 secreted into supernatant culture medium accumulated at concentrations up to 246.53 ± 15.92 pg.mL ^−1^ .

## DISCUSSION

Although human knowledge is aware of how the host’s immune system controls growth of Leishmania, production of an effective vaccine against this chronic disease is one of the great scientific challenges. Cytokines are an inseparable part of the intercellular communication network required for start and control of immune response (17). IL-12 is a heterodimer cytokine consisting of two subunits: p35 and p40 (18). Necessity of the proper folding between IL-12 subunits is one of the challenges in expressing of the recombinant protein form. To overcome this challenge, many engineered strategies of gene structures have been utilized. For instance, many studies have been performed in this field such as, using T7 promoter and EMCV IRES between two sections of hIL-12 p35, p40 in measles virus in Vero cells as a host (19), hCMV promoters for p35 and sCMV promoter for hIL-12 p40 in HEK293 cells and use of Cauliflower Mosaic Virus (CaMV) promoter before hIL-12 p40, p35 subunits in plant cells to express interleukin 12 (20). To improve expression and secretion of human recombinant IL-12 protein, two strategies were used in this study: specific Leishmania signal peptide and CMV promoter. In many studies, the common vector for gene transferring into *Leishmania tarentolae* rDNA is pLEXSY (21–23). In the present study, I.L.L was used to express recombinant hIL-12 and targeted gene inserted into CPC site using homologous recombination. CPC is considered as one of the pathogenic parasite genes and destruction of that does not cause the disturbance parasite’s life cycle.

The results of this study showed that the designed vector, pKDB-cpc can be utilized as a new carrier to transfer gene into I.L.L genome.

## CONCLUSION

Targeted gene replacement into the I.L.L-CPC gene using plasmid pKDB-cpc identical replacement process was successfully completed for the first time. Stabilized recombinant DNA consist of target gene did not show any toxicity for the parasite. Transgenic I.L.L produced and secreted active human interleukin 12. Recombinant hIL-12 could be utilized as an adjuvant in completion and stimulation of cellular immunity response. Product of this study can be an appropriate candidate for Leishmanization.
